# Fe(III) doped carbon nanodots with intense green photoluminescence and dispersion medium dependent emission

**DOI:** 10.1038/s41598-019-55264-x

**Published:** 2019-12-11

**Authors:** Corneliu Sergiu Stan, Adina Coroabă, Elena Laura Ursu, Marius Sebastian Secula, Bogdan C. Simionescu

**Affiliations:** 1 Gheorghe Asachi Technical University of Iasi, Faculty of Chemical Engineering and Environmental Protection, Iasi, 700050 Romania; 2 Petru Poni Institute of Macromolecular Chemistry, Department of Chemistry, Iasi, 700487 Romania

**Keywords:** Nanoparticles, Nanoparticles, Nanoparticles, Nanophotonics and plasmonics

## Abstract

The preparation and investigation of Fe(III) doped carbon nanodots (CNDs) with intense green photoluminescence and emission dependence on the dispersion medium are reported. Their unusual photoluminescence is especially highlighted in water where the initial blue emission is gradually shifted to intense deep green, while in other common solvents (chloroform, acetone etc.) this behavior has not been observed. Through embedding in a polymer matrix (e.g., PVA) the color transition becomes reversible and dependent on water content, ranging from a full blue emission, when completely dried, to an intense green emission, when wetted. The preparation path of the Fe(III) doped CNDs undergoes two main stages involving the initial obtaining of Fe(III)–N–Hydroxyphthalimide complex and then a thermal processing through controlled pyrolysis. Morphostructural investigations of the prepared Fe(III) doped CNDs were performed through TG, FT-IR, XPS, DLS, TEM and AFM techniques whereas absolute PLQY, steady state and lifetime fluorescence were used to highlight their luminescence properties. The results issued from structural and fluorescence investigations bring new insights on the particular mechanisms involved in CNDs photoluminescence, a topic still open to debate.

## Introduction

CNDs are a new class of nanostructured materials which gathered an increasing attention during the last decade due to their particular features such as excitation dependent photoluminescence, resistance to photobleaching, biocompatibility and lack of toxicity, facile surface functionalization or dispersibility in various solvents etc^1,2^. By definition, the CNDs structure consists of a carbonaceous core with a defect rich graphitic configuration which is surface decorated with various functional groups^3^. Although many studies focused on their implementation in different application areas including sensors^4^, bioimaging^5^, drug delivery^6^, catalysis^7^, optoelectronics^8^, controversies on their structural configuration or photoluminescence mechanisms still exist^9,10^. Their excitation dependent photoluminescence is a particular feature, which triggered much debate as concerns the mechanisms involved in the radiative processes. A first approach is based on a behavior similar to that of semiconductor Quantum Dots, where the emission peaks are size dependent and rely on quantum confinement^11^ while the second one highlights the radiative transitions occurring within or between the surface attached functional groups, with the graphitic core defects playing a key role over the entire process^12^. The latter approach seems to gain more evidence as very recent studies revealed the importance of carbonyl or nitrogen containing groups and also the influence of the dispersion medium on the emission characteristics of the CNDs^13,14^. In most cases, the photoluminescence (PL) emission is located in the blue region of the visible spectrum irrespective of the starting precursors. However, an increasing number of papers reported green, yellow or even red emission^15–17^. Usually, the emission located in the inferior regions of the visible spectrum is achieved through surface modifications yet, with few exceptions^18,19^, for the price of a lower intensity. CNDs preparation methods could be roughly divided into two main categories: top-down approaches, where bulk carbon materials like graphite, graphite oxide or carbon soot are processed through combustion, arc discharge, laser ablation or electrochemical routes^20^ and bottom-up approaches, where various organic compounds are used as precursors in pyrolytic, hydrothermal and microwave/ultrasonic assisted processes^21^. The doping of CNDs^22^ with various elements including nitrogen^23^, phosphorus^24^, sulfur^25^, boron^26^ or transition metals (Zn, Mn)^27,28^ can alter the emission spectra while the overall efficiency of the radiative processes can be markedly enhanced. In the present paper, new highly green emissive Fe(III) doped CNDs are reported. Fe(III)-doped CNDs were prepared through a straightforward pyrolytic^29^ processing of a Fe(III)–N–Hydroxyphthalimide intermediate complex. The achieved CNDs present an unusual reversible emission transition when dispersed in water, the emission turning gradually from deep blue to intense green in the presence of ambient light or exposed to UV (370 nm). Their unique feature of emission transition reversibility was tested by embedding the Fe(III) doped CNDs in a polymer matrix (PVA in this case, though not limited to) leading to color transition depending on water content, turning from a full blue emission when completely dried to intense green when wetted. To our knowledge, the reported CNDs are the first to exhibit this type of behavior, opening several new perspectives in terms of potential applications and providing several new insights regarding their specific structure and luminescent mechanisms.

## Results and Discussion

**Figure 1:**
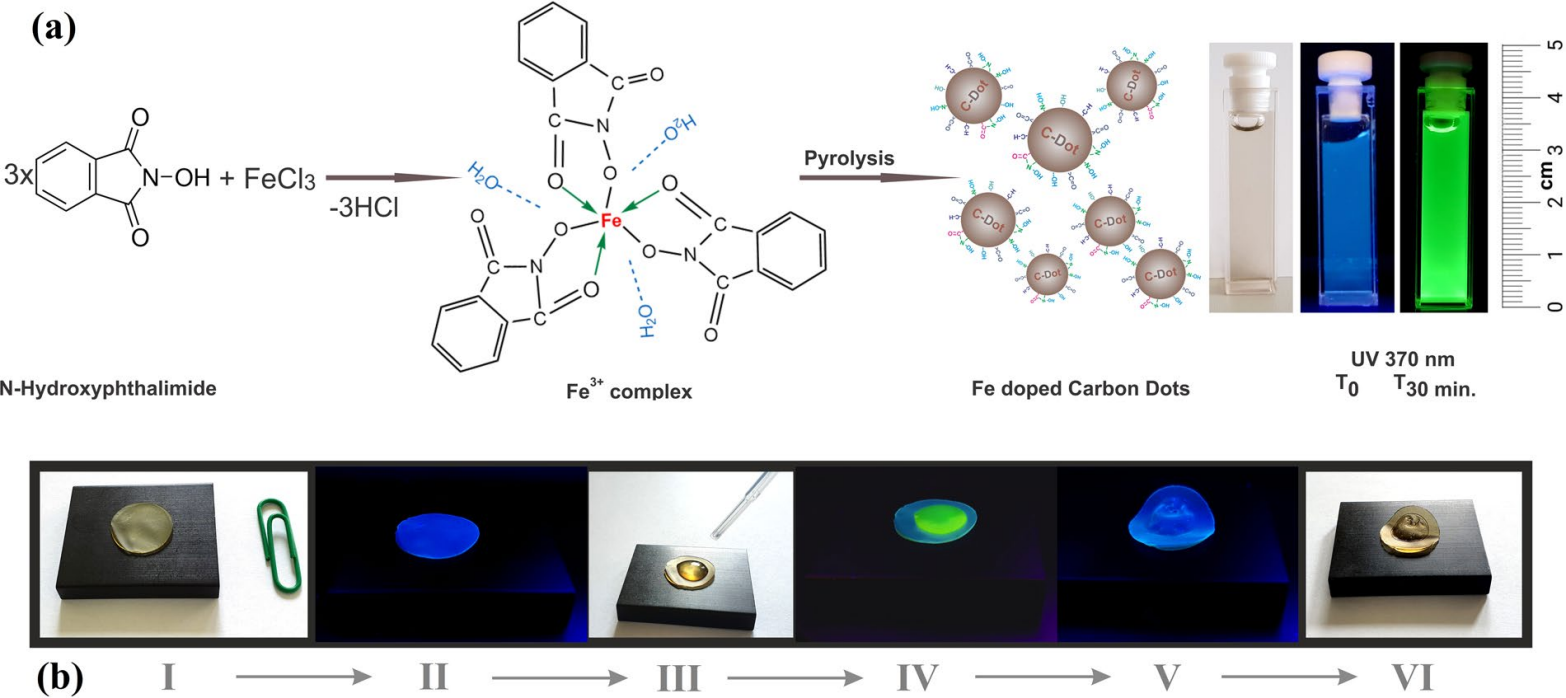
(**a**) Preparation stages of the Fe doped CNDs: intermediary prepared complex, resulted CNDs under ambient light and UV (370 nm) radiation, respectively; (**b**) Sequence of the reversible emission shift from blue to green of the PVA-Fe doped CNDs composite.

Figure 1a presents the preparation stages of the Fe(III) doped CNDs. As can be seen, in the first stage Fe(III) is complexed with N–Hydroxyphthalimide at 1:3 metal to ligand ratio, resulting in a complex with the suggested structure sustained by TG, FT-IR and XPS investigations. The $[Fe{({C}_{8}{H}_{4}N{O}_{3})}_{3}{({H}_{2}O)}_{x}]$ complex is the result of the bidentate behavior of the N–Hydroxyphthalimide ligand. The coordination number is 6, which is typical for Fe(III)^30^, the covalent bonding being established between the Fe(III) cation and the oxygen in the –O–N< group while a coordinative bonding occurs between the cation and the oxygen in the carbonyl group –O=C<^31^. Water molecules are also present in the inner/outer coordination sphere. In the second stage the prepared complexes processed by pyrolysis result in water-dispersed CNDs as described in Preparation subsection. Further, the Fe(III) doped CNDs were introduced in a PVA matrix. Figure 1b shows the sequence of the emission shift from blue to green of the Fe doped CND embedded in PVA (I- prepared composite under ambient lighting, II- initial blue region located emission under 370 nm UV radiation, III-H_2_O wetting of the composite, IV- shifted blue to green emission of the wetted area after 20–30 min, V- oven re-dried composite reversed blue emission, VI- aspect of the re-dried composite under ambient lighting).

### Thermal analysis

Thermal analysis of the intermediate complex provided useful information on its structural configuration and expected thermal behavior during the second stage pyrolytic processing leading to CNDs formation. The recorded decomposition stages are detailed in Table 1 while Fig. S1 presents the mass loss variation within the investigated interval. In the first stage, as suggested by the recorded value, water molecules located within the inner or outer coordination sphere are lost. The steep mass loss recorded in the second decomposition stage is most probably due to the advanced destructuration of the complex which is accompanied by volatiles exhaustion. This stage is particularly important from the perspective of the pyrolytic process leading to CNDs. The experimentally established pyrolysis parameters (210 °C, 6 min) allow the formation of the graphitic core yet, also through partial destructuration, various functional groups still remain available within the final configuration of the Fe(III) doped CNDs. In the 3^*rd*^ and 4^*th*^ stages, the decomposition processes are still present, but to a smaller extent, while between 402–900 °C no mass loss was recorded, the final decomposition residue being attained.

**Table 1:** Decomposition stages recorded for the Fe(III)–N–Hydroxyphthalimide complex.

Stage 1		Stage 2		Stage 3		Stage 4		Residue
ΔT, (°C)	Δm, (%)	ΔT, (°C)	Δm, (%)	ΔT, (°C)	Δm, (%)	ΔT, (°C)	Δm, (%)	(%)
25–122	14.56 ± 0.18	168.6–250	44.81 ± 0.19	250–331	19.01 ± 0.15	357–402	6.46 ± 0.19	15.16 ± 0.21

### FT-IR analysis

**Figure 2:**
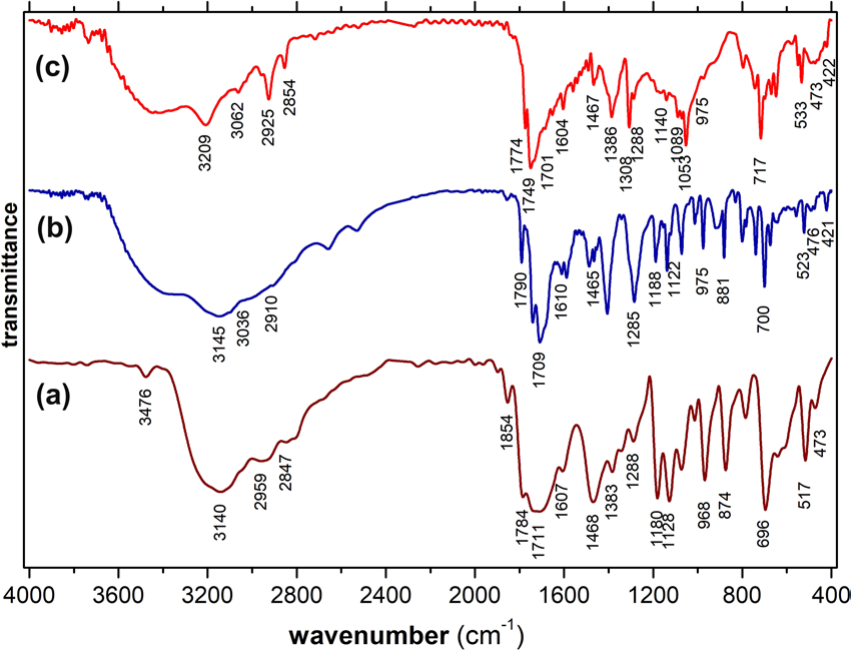
IR spectra recorded for (**a**) N–Hydroxyphthalimide ligand, (**b**) Fe(III)–N–Hydroxyphthalimide complex and (**c**) Fe(III) doped CNDs.

IR spectra (Fig. 2) were acquired in case of both the intermediary Fe(III)–N–Hydroxyphthalimide complex and the resulted Fe(III) doped CNDs. To highlight the modifications occurring through complexation, the spectrum of N–Hydroxyphthalimide ligand was also recorded.

Table S1 lists several significant specific vibrations of various groups^32^ and highlights the modifications occurring through complexation and further pyrolytic processing of the complex, leading to Fe(III) doped CNDs. The formation of the complex (Fig. 2, line (b)) is illustrated by a series of modifications, as follows: the intense characteristic band with a maximum peak at 1784 cm^−1^ corresponding to the sym. carbonyl (O=C<) stretch appearing for the ligand is shifted to 1740 cm^−1^ in case of the complex, as a result of the coordinative bonding established between the central Fe^3+^ cation and the oxygen atoms; the new peak occurs at 421 cm^−1^ due to the stretching vibration of the newly established Fe–O–N< bonding while the –OH stretch peak is no more present. Due to the rearrangements determined by the complexation, other vibrations corresponding to the aromatic and succinic rings are shifted toward higher wavenumbers as a result of the influence of the central Fe(III) cation. As for the Fe(III) doped CNDs (Fig. 2, line (c)), the structural modifications resulted following the partial pyrolysis are clearly visible in the recorded spectrum. Thus, in the upper region (3300–2800 cm^−1^) the recorded peaks corresponding to C–H, C–C and C=C groups are displaced and more intense, while in the mid and lower regions a series of peaks corresponding to various groups are less intense, displaced or missing compared to the Fe(III)–N–Hydroxyphthalimide complex. These modifications occur due to the formation of the carbonaceous core and the re-arrangements of the functional groups within the defect rich graphitic structure or surface attached as terminal groups. The characteristic band at 421 cm^−1^, corresponding to the Fe–O– stretch vibration is still present in the recorded spectrum but its intensity is markedly lower. The thermal processing parameters (temperature and duration of the thermal exposure) play a key role in achieving the favorable configuration of the resulted Fe(III) doped CNDs. During the experimental study on the optimal pyrolytic processing temperature and duration of the main sequence of the partial decomposition process, it was observed that higher temperature values or longer thermal exposure lead to a severe diminution in the emission most probably due to an enhanced depletion of the attached functional groups.

### XPS analysis

**Figure 3:**
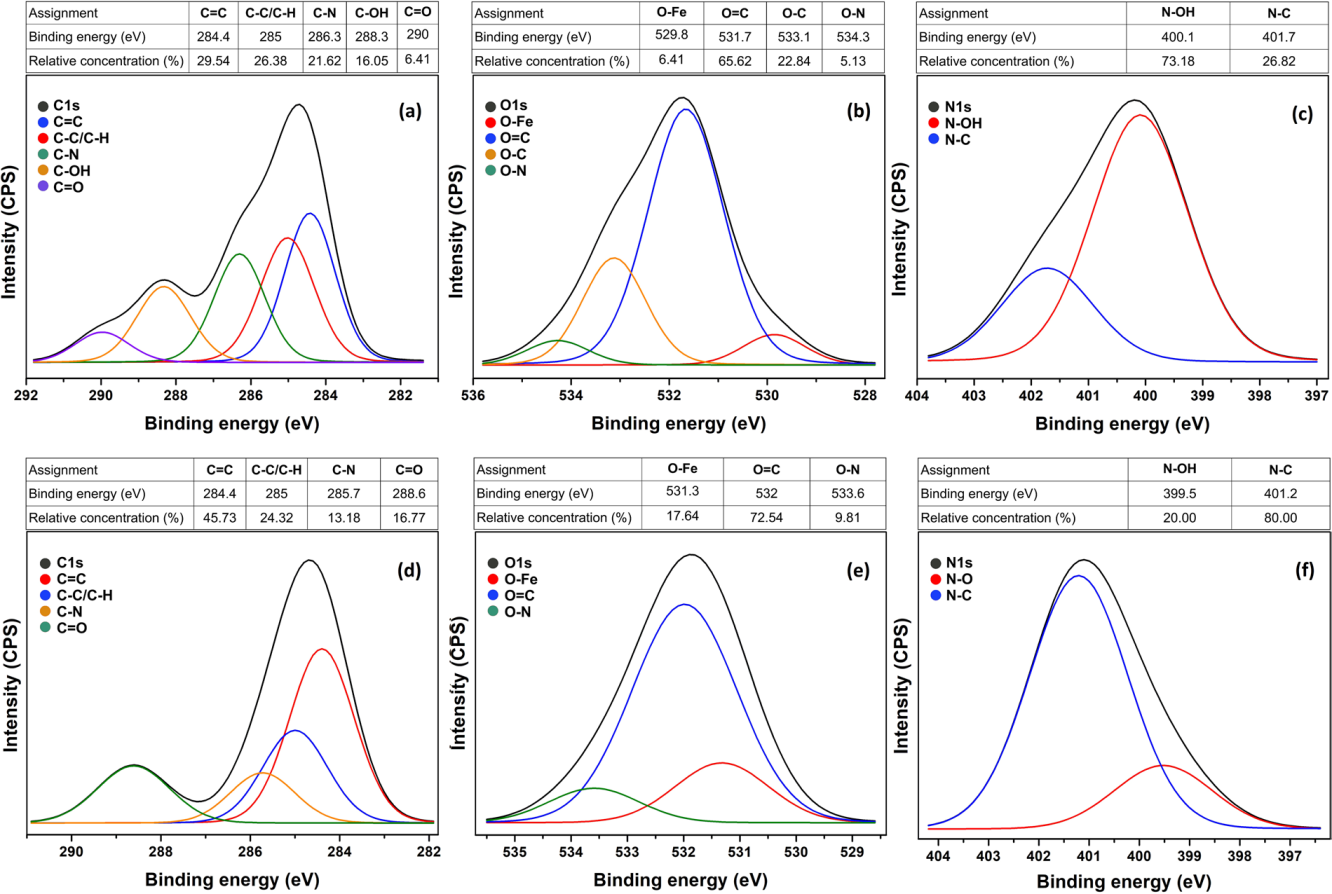
High resolution spectra and relative concentrations of various groups recorded for Fe(III) complex (**a**) C1s, (**b**) O1s and (**c**) N1s, and for Fe(III) doped CNDs (**d**) C1s, (**e**) O1s and (**f**) N1s.

XPS investigation revealed the relative concentrations of various functional groups in both the prepared Fe(III) complex and Fe doped CNDs. Figure 3a–c provide the high resolution spectra (C1s, O1s, N1s) recorded for the Fe(III) complex, while the relative concentrations of main functional groups are detailed in the attached tables. The results are in good agreement with the proposed structure of the Fe(III) complex. The Fe–O bonding achieved through complexation is clearly visible and in agreement with the FT-IR investigation. Figure 3d–f present the high resolution spectra recorded for the Fe(III) doped CNDs along with the relative concentrations of remnant functional groups. As could be noted from the C1s spectrum, the increased values of the C=C groups are the result of the carbonaceous core issued through the pyrolytic processing. The presence of C–C/C–H bonds suggest a highly disordered, defect rich graphitic structure. The relatively high concentrations of C=O, C–N, N–O groups is also an indication of various remnant groups still attached to the carbonaceous core, which most probable are responsible for the radiative transitions leading to the observed PL properties. The Fe–O bonding is still present in even higher concentrations as compared to the complex as a result of the volatiles depletion occurring during the pyrolytic processing. Fit residual plots are detailed in Fig. S2.

### Dimensional investigation

**Figure 4:**
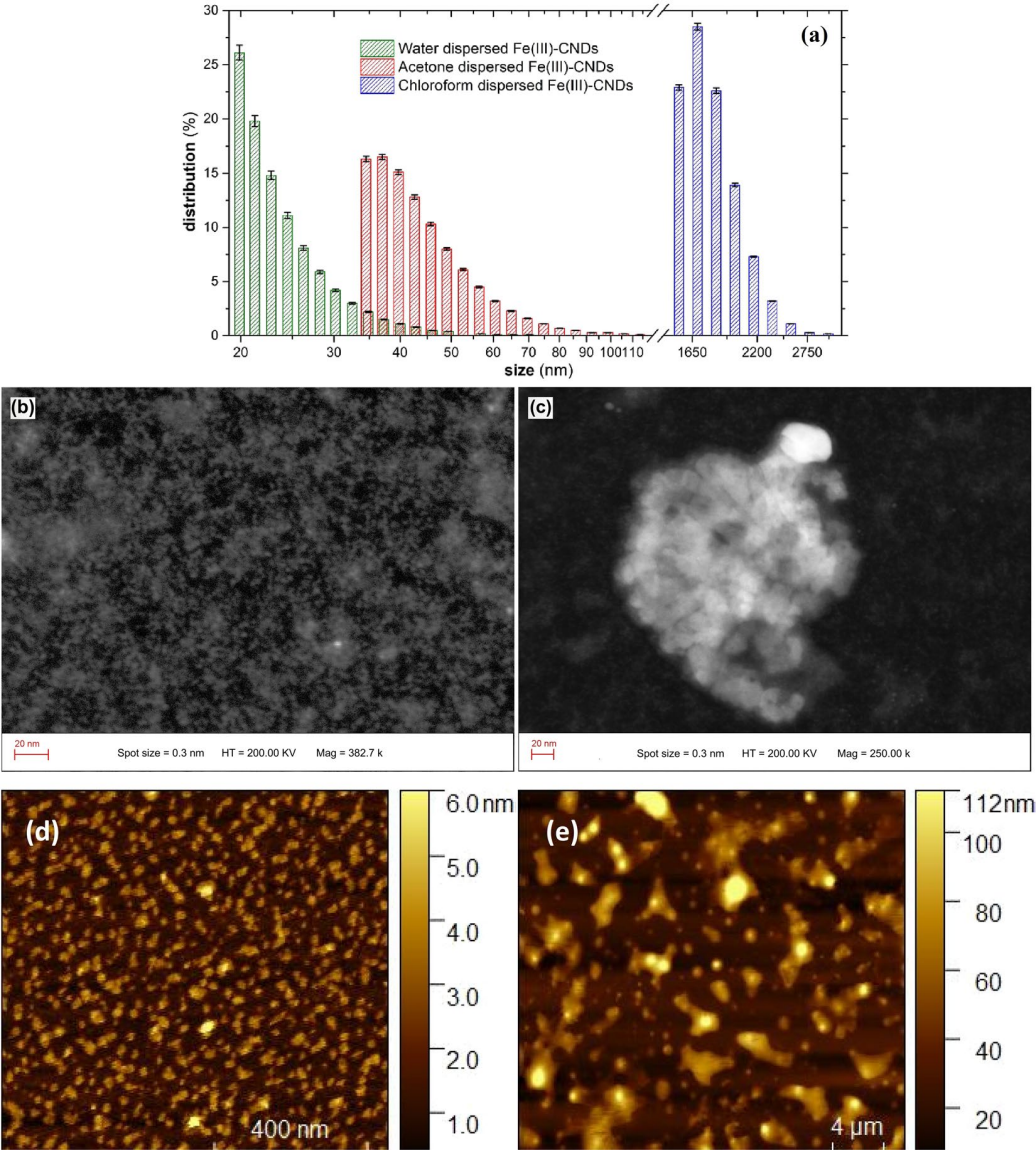
Dimensional distribution of the prepared Fe(III) doped CNDs (**a**) dispersed in water, acetone and chloroform; TEM micrographs (**b,c**) of Fe(III) doped CNDs; and AFM imaging recorded for Fe(III) doped CNDs dispersed in (**d**) water, (**e**) chloroform.

For the dimensional investigation, Fe(III) doped CNDs were dispersed in three solvents. Thus, water (polar protic), acetone (polar aprotic) and chloroform (non-polar)^33^ dispersions were prepared by suspending 1 mg of Fe(III) doped CNDs in each 10 mL of the ante-mentioned solvents, the mixtures being further sonicated and then centrifuged at 15000 RPM for 10 min. The recorded results are presented in Fig. 4a. In aqueous medium the Fe(III) doped CNDs are well dispersed, with 20–25 nm average size of individual dots or clusters representing about 60%, while the rest are up to 35 nm. The situation is slightly different in acetone, the dimensional distribution between 35–50 nm in a proportion of 75%, the rest being at a max. 60–120 nm range. For chloroform dispersed Fe(III) doped CNDs the agglomeration tendency is very high, with clusters exceeding 1.5 *μ*m. The average recorded measurement errors for the Fe(III) doped CNDs dispersed in water, acetone and chloroform, respectively, are: 2%; 1.5%; 1.1%. The results confirm the significant influence of the dispersion medium on the agglomeration tendency of the CNDs^7,34^. In the particular case of aqueous dispersion, the Fe^3+^ cation seems to favor the hydration of the individual CND. The presence of water molecules in the outer coordination sphere or interacting with the surface located functional groups seems to avoid the agglomeration tendency to a certain extent. As will be later shown, the presence of water molecules is responsible for both unusual color shift and deep green photoluminescent emission, while for the other solvents this behavior is absent.

### TEM investigation

The recorded TEM micrographs also revealed comprehensive information regarding the size and clustering tendency of the Fe(III) doped CNDs. As could be noted from Fig. 4b, the size of the nanostructures ranges from 2–3 nm to hundreds of nm. The larger entities are the result of an agglomeration process, a granular structure being clearly visible. The smaller entities in 2–3 nm range could be individual CNDs, yet a clustered structure is also possible. Figure 4c clearly reveals the granular structure of a larger aggregate (120–140 nm), smaller clustered entities (15–25 nm) being also visible.

### AFM investigation

AFM imaging provided useful information regarding the different agglomeration tendency as a function of the solvent used as dispersion medium. Images were recorded for water and chloroform solutions (prepared as described in the prior section) deposited through evaporation on mica substrates. Figure 4d shows the image recorded for the Fe(III) doped CNDs dispersed in water. As can be observed, the average particle size is in the 20–40 nm range, which is in a very good agreement with DLS results. The dimensional distribution is narrow and the dried nanostructures are evenly spread on mica substrate. As for sample prepared from Fe(III) doped CNDs dispersed in chloroform (Fig. 4e), the average particle size is in 2–3 *μ*m range, also in very good agreement with DLS results. The size distribution is markedly larger as compared to the aqueous dispersion, while the cluster shapes are irregular.

### Fluorescence investigations

Photoluminescence (PL) of the CNDs is one of their most interesting and debated properties. Usually, they exhibit a blue, excitation dependent emission when dispersed in various media (solvents, polymer matrices etc.). The intensity and emission peak positions are also closely dependent on the type of the dispersion medium^35^. As mentioned in the first section, the PL mechanism is still an actively debated subject, with an approach which emphasizes a size-depending emission under quantum confinement conditions and alternatively, the preponderant role of structural defects within the carbonaceous core/surface attached functional groups and interactions occurring between or within various constituent atomic species^36,37^.

#### Steady state fluorescence investigation

**Figure 5:**
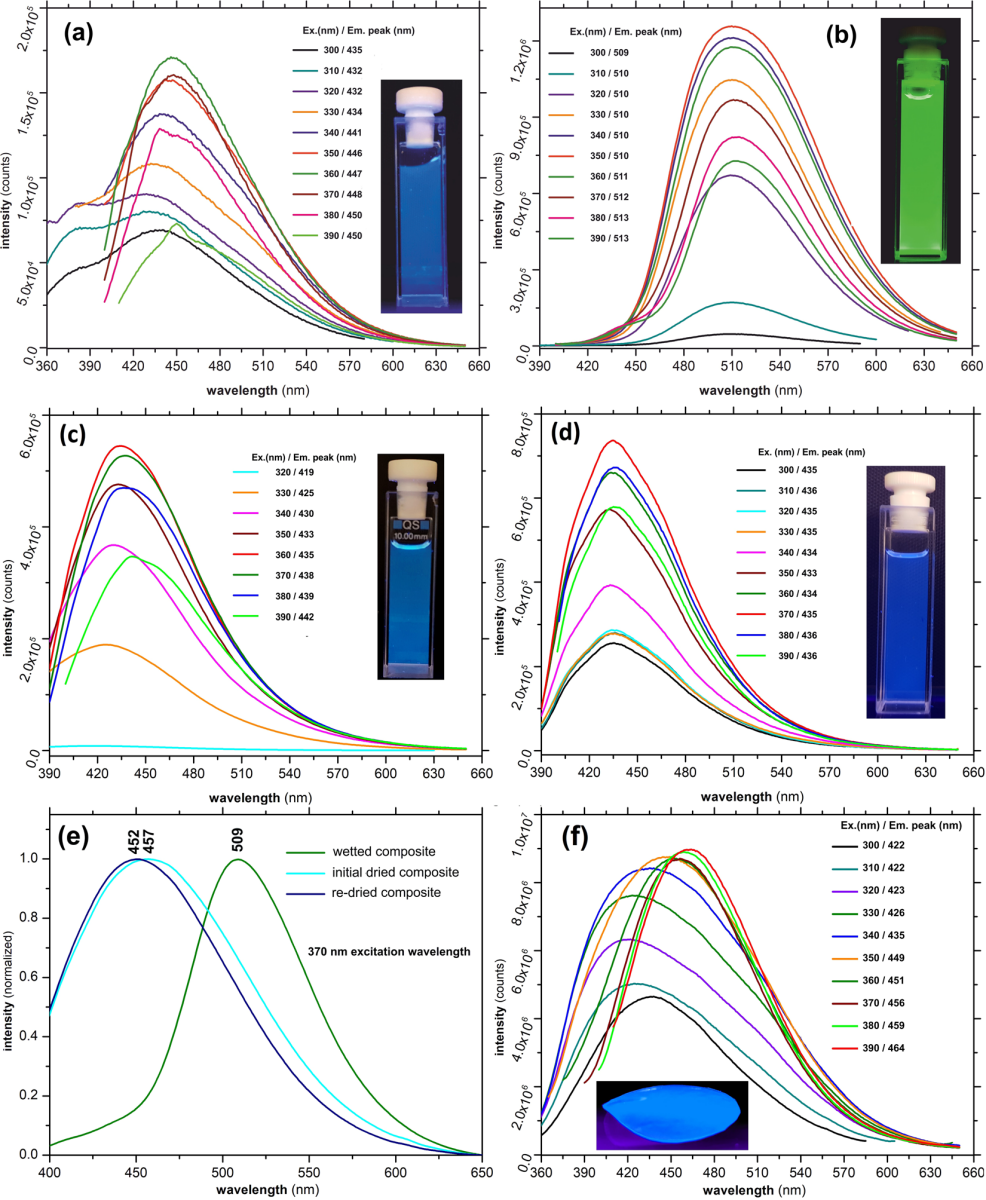
Emission spectra recorded for the Fe(III) doped CNDs dispersed in: (**a**) water (freshly dispersed); (**b**) water - after 30 min of UV exposure; (**c**) acetone; (**d**) chloroform; (**e**) emission shifting of the PVA- Fe(III) doped CNDs composite and (**f**) for the PVA- Fe(III) doped CNDs composite.

As previously mentioned, the water dispersed Fe(III) doped CNDs present some interesting particularities of their emission. Following the preparation and dispersion in water, the emission is initially located in the blue region of the visible spectrum but, under UV exposure (as well as under ambient solar light), it gradually turns to intense green, which further remains indefinitely stable. The color transition occurs within 30–35 min after initial water dispersion and sunlight/UV radiation exposure. Figure 5a presents the PL emission spectra recorded immediately after water dispersion/UV exposure while the spectra in Fig. 5b were recorded after 30 min.

**Figure 6:**
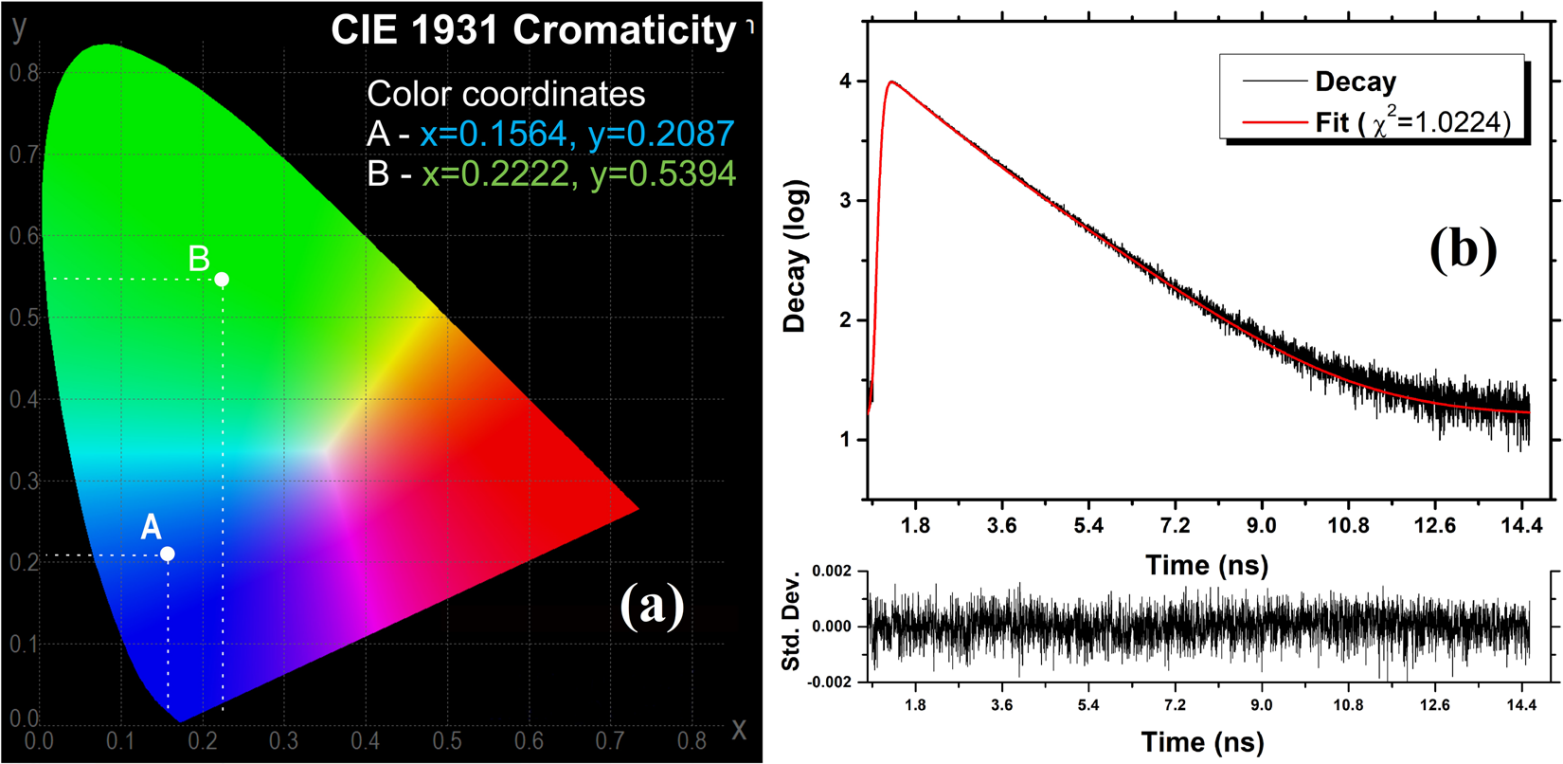
CIE 1931 chromaticity of water dispersed Fe(III) doped CNDs (**a**) and fluorescence lifetime of water dispersed Fe(III) doped CNDs (**b**).

As can be noted, in both cases the luminescent emission is excitation dependent within the investigated interval (300–390 nm in 10 nm steps) with emission peaks occurring in 435–450 nm range and 509–513 nm, respectively. The maximum emission intensity peaks were observed at 447 nm (360 nm excitation) and at 510 nm (350 nm excitation), respectively. Visually, the intensity emission is markedly higher in case of green emission achieved after 30 min. compared to initial blue emission observed prior to light exposure. Figure 5c,d present the recorded spectra of the Fe(III) doped CNDs dispersed in acetone and chloroform. In both cases the PL emission is located in the blue region of the visible spectrum and remains unchanged (both peaks location and intensity) for an indefinitely long exposure to UV. Thus, in case of acetone dispersion, the peaks were recorded within 419–442 nm range (320–390 nm excitation, 10 nm steps) while for the chloroform dispersion, the peaks are almost excitation independent (434–436 nm within 300–390 nm excitation range). In both solvents the emission intensities are comparable, yet slightly lower than those recorded for the freshly water dispersed/UV exposed Fe(III) doped CNDs (Fig. 5a). The recorded results lead to several interesting conclusions concerning the mechanism involved in the transition from blue to intense green emission. Water molecules seem to play a key role along with the particular structural configuration resulting due to the presence of Fe(III) cations. While a series of other prepared N–Hydroxyphthalimide transition metals (Mn, Co, Ni) complexes were also tested during the preliminary experiments, no comparable results were obtained. The presence of the Fe(III) cations attached to the remnant surface located groups or entrapped as defects within the carbonaceous core affect the radiative transitions occurring within various functional groups or within the traps created in the defect rich structure of the core. Not to be neglected, the Fe(III) cation could favorably influence the local emissive sites through its particularities such as ionic radius for instance, to influence positively the local emissive sites. Also, through hydration, the present OH groups tend to alter the excited states and the resulted photonic emission^38,39^. Most probably, during the mentioned 30 min interval, water molecules gradually migrate toward outer coordination sphere of the Fe(III) cations, arriving in the vicinity of the groups responsible for the radiative transitions. The presence of the OH oscillators seems to favor the local interactions occurring within or between various functional groups or atomic species essentially involved in achieving the excited states responsible for the photonic emission. The blue to green emission shift is also a possible consequence of the OH oscillators presence near the emission sites, the resulted photons having a lower energy (higher Stokes shift). To test the assumption that the blue to green emission shifting is mainly a result of water molecules presence, the Fe(III) doped CNDs were introduced in a PVA matrix according to the procedure described in preparation subsection. The transparent, light yellow tinted aspect of the resulted polymer composite film, under ambient lighting conditions is presented in Fig. S2. When completely dried, the film presents a relatively intense blue emission under UV. As expected, when wetted or placed in a high humidity environment, the composite PL gradually turns to a green emission which further returns to a blue emission after drying, the process being reversible as presented in the recorded photo sequence detailed in Fig. 6a. The reversible emission shifting is highlighted by the recorded emission spectra presented in Fig. 5e. Figure 5f presents the emission spectra of freshly prepared and dried PVA-Fe(III) doped CNDs composite. As can be noted, the recorded emission spectra also presents the specific excitation dependent emission, with peaks located within 422–464 nm range (300–390 nm excitation, 10 nm steps). Compared to the freshly dispersed Fe(III) doped CNDs (Fig. 5a) the emission peaks are slightly displaced to lower wavelengths, most probably due to the influence of the polymer matrix^40,41^ or presence of remnant water content within the composite. As a conclusion, the above mentioned results inherently support the PL mechanism which emphasizes the preponderant role of the surface attached functional groups and/or defects located within the carbonaceous core. As issued from the experimental, the dimensional characteristics of the CNDs are hardly to be linked to the observed behavior.

#### PLQY, chromaticity and lifetime investigations

The absolute PLQY investigations were performed in the 310–390 nm excitation range (10 nm steps) for the Fe(III) doped CNDs dispersed in three types of solvents (water - polar protic, acetone - polar aprotic and chloroform - non-polar), the results being presented in Table 2. The highest PLQY value (33.12%) is achieved at 330 nm excitation in case of water/after 30 min interval, while for the freshly water dispersed/unexposed sample a 26.81% value (350 nm excitation) was recorded. The acetone dispersed Fe(III) doped CNDs achieved a 28.04% PLQY at 320 nm excitation. For the chloroform dispersion, the max. PLQY (29.47%) was recorded at 390 nm excitation.

**Table 2:** Recorded PLQY for the Fe(III) doped CNDs, and their corresponding absolute error values.

Fe(III) doped CNDs dispersed in:	Excitation wavelength (nm)								
	310	320	330	340	350	360	370	380	390
	PLQY (%)								
water (freshly dispersed)	16.14 ± 0.06	20.18 ± 0.06	23.44 ± 0.06	25.96 ± 0.06	**26.81** ± 0.08	25.62 ± 0.09	24.01 ± 0.08	21.06 ± 0.07	18.73 ± 0.06
water (after 30 min UV exp.)	25.73 ± 0.02	25.08 ± 0.02	**33.12** ± 0.03	32.66 ± 0.02	30.94 ± 0.02	25.58 ± 0.01	20.19 ± 0.01	17.14 ± 0.01	16.55 ± 0.01
acetone	26.57 ± 0.20	**28.04** ± 0.24	27.10 ± 0.16	25.31 ± 0.20	22.66 ± 0.16	19.14 ± 0.20	16.23 ± 0.20	13.09 ± 0.10	10.38 ± 0.10
chloroform	20.01 ± 0.03	21.16 ± 0.03	21.37 ± 0.04	22.52 ± 0.04	25.56 ± 0.08	26.75 ± 0.09	28.84 ± 0.13	28.76 ± 0.13	**29.47** ± 0.22

As can be seen, for both types of polar solvents the highest values were recorded in 320–350 nm (within UV-B) excitation range while for the non-polar solvent the maximum PLQY was achieved in upper UV range (UV-A). The recorded results suggest interactions occurring between solvent and emissive centers, in case of polar solvents, higher energy UV excitation photons being required to trigger the radiative transitions. Figure 6a presents the chromatic parameters according to CIE 1931 color space, recorded for water dispersed Fe(III) doped CNDs with initial bluish emission (A) and shifted greenish emission achieved after 30 min. (B).

Fluorescence lifetime of the radiative processes occurring in water dispersed Fe(III) doped CNDs is presented in Fig. 6b. While the overall lifetime is located in nanoseconds range, several interesting details were highlighted during investigation. Similar to what have been found for other CNDs, the fluorescence lifetime decays could not be deconvoluted with a mono-exponential function^42,43^. The deconvolution fits became satisfactory with the use of a tri-exponential function, for which the corresponding pre-exponential factors (A_1_, A_2_ and A_3_) and lifetimes (${\tau }_{1}$, ${\tau }_{2}$ and ${\tau }_{3}$) are shown in Table S2. These values were used to calculate the average fluorescence lifetime according to the equation <${\tau }_{F}$> = [(A_1_(${\tau }_{1}$)^2^ + (A_2_(${\tau }_{2}$)^2^ + (A_3_(${\tau }_{3}$)^2^]/(A_1_${\tau }_{1}$+A_2_${\tau }_{2}$ + A_3_${\tau }_{3}$)^44^. The recorded data supports the PL mechanism which is related to the radiative transitions occurring within/between surface attached groups or within the traps located in the defect rich graphitic structure of the carbonaceous core.

## Experimental

### Materials

N–Hydroxyphthalimide (97%), anhydrous FeCl_3_ and Poly(vinyl alcohol) (PVA) Mw = 58000 were sourced from Merck Chemicals. High purity water and reagent grade ethanol (EtOH) were used for preparation of the Fe(III)–N–Hydroxyphthalimide complex, intermediate purification stages and dispersion, while reagent grade acetone, chloroform, EtOH provided by the same supplier were used as re-dispersion mediums for the prepared CNDs.

### Preparation

In the first stage a Fe(III) - N–Hydroxyphthalimide complex was prepared at 1:3 metal to ligand ratio. In a typical procedure 0.5 g anhydrous FeCl_3_ was dissolved in 10 mL of water while in a separate glass beaker 1.5 g N–Hydroxyphthalimide was dissolved under magnetic stirring in a mixture of 20 mL water and 25 mL EtOH at a moderate 45–50 °C temperature. The two resulted solutions were mixed under continuous stirring and kept overnight at 35–40 °C for reaction completion. The resulted dark brown precipitate was washed at least three times with water and further dried under vacuum. In the second stage the powder of fine milled complex was processed in the experimental setup presented in Fig. S3. The flowchart of the preparation stages are detailed in Fig. S4. The experimental set-up consists of a quartz tube, a heating mantle, a temperature/flow controlled hot air source along with a water container, an evacuation pump and the required piping. Thus, 0.3 g of Fe(III) - N–Hydroxyphthalimide complex was added in the quartz tube and heated at 210 °C for 6 min in N_2_ atmosphere. Following the completion of the pyrolytic process, the reaction product was flooded with cold water (3–5 °C). The resulted CNDs aqueous dispersion was evacuated and further centrifuged at 15000 RPM for about 10 min. The aqueous supernatant is again centrifuged in the same conditions, the collected supernatant containing dimensionally selected Fe(III) doped CNDs was used as obtained or freeze dried for re-dispersion in other solvents (ex. acetone, chloroform). The aqueous dispersion of the prepared Fe(III) doped CNDs presents a slightly yellow tinted transparent aspect under normal illumination conditions. When exposed to solar light or UV radiation (370 nm) immediately after preparation, the dispersion initially emits in deep blue which gradually (within 30 min) turns to intense green emission (Fig. 1b). The polymer film composite is prepared by dissolution under stirring of 1 g PVA in 15 mL of previously obtained aqueous dispersed Fe(III) doped CNDs and further drying in ambient conditions in a conveniently shaped mold. The transparent, light yellow tinted aspect of the resulted polymer composite film, under ambient lighting conditions is presented in Fig. S5.

### Characterization methods

Structural investigations were performed for both the prepared Fe(III)–N–Hydroxyphthalimide complex and Fe(III) doped CNDs. Quantitative data are expressed as the mean value +/− standard deviation. Prior to analysis, the samples were vacuum oven dried at 60 °C for 12 h. The thermal analysis of the prepared complex was performed in triplicate using a Mettler Toledo TGASDTA851e instrument working in N_2_ atmosphere with 10 °C/min heating rate within the 25–900 °C range. The FT-IR spectra were recorded in duplicate in the 400–4000 cm^−1^ range using a Shimadzu IRAffinity 1S spectrometer according to KBr method. Dimensional analysis and size distribution (performed in triplicate) of the Fe(III) doped CNDs dispersed in water, acetone and chloroform were carried out on a Beckman-Coulter Delsa Nano. Prior to investigation the solutions were twice centrifuged at 15000 RPM for 10 min. The AFM investigations were performed using a Ntegra Spectra (NT-MDT, Russia) instrument provided with silicon cantilever tips (NSG 10) operated in tapping mode under ambient conditions. The Fe(III) doped CNDs samples were deposited on mica substrates from high diluted water and chloroform solutions. TEM images were recorded on a Zeiss Libra 200MC UHR-TEM working at 200 KV acceleration voltage and a 0.3 nm spot size. The samples were deposited on carbon grids from a diluted EtOH solution. The XPS spectra were obtained on a ULVAC-PHI, 5000 VersaProbe spectrometer (Physical Electronics), equipped with a monochromatic Al K*α* X-ray source (h*v* = 1.486 keV). During measurements, the pressure in the analysis chamber was maintained at 5.9 × 10^−8^ Pa and the photoelectron take-off angle relative to the sample surface was 45°. The binding energy was calibrated by the peak energy of C 1 s (284.6 eV) as reference and the resolution of the XPS analyzer is 0.85 eV for organic materials. The XPS data were analyzed using XPSPeak41 software for peak deconvolution. The Steady-State Fluorescence, Time-Correlated Single-Photon-Counting (life-time), absolute PLQY and chromaticity parameters measurements were conducted on a Horiba FluoroMax-4 equipped with Quanta Φ integration sphere for absolute PLQY measurements, TCSPC lifetime module and adapter for solid state sample investigations. The steady state fluorescence, PLQY and chromaticity parameters were acquired with the FluorEssence software according to the manufacturer recommended procedures. Lifetime measurements were performed according to the manufacturer procedures using the provided DAS6 Decay Analysis Software. The investigation was performed in freshly prepared diluted samples according to the measurements requirements.

## Conclusions

Fe(III) doped CNDs with intense green photoluminescence and unusual emission dependence according to the dispersion medium were prepared through controlled pyrolytic processing of a Fe(III)–N–Hydroxyphthalimide complex. Their unusual photoluminescence is especially highlighted in water where the initial blue emission is gradually shifted to intense deep green. When embedding the prepared CNDs in a PVA polymer matrix, the color transition was found to be reversible and dependent on water content. The structural investigation performed through TG, FT-IR, XPS revealed a typical configuration consisting of a carbonaceous core which is highly surface decorated with various functional groups. DLS, TEM and AFM were used to investigate their morphology. The dimensional distribution is solvent dependent and due to the interactions occurring between surface located groups a clustering tendency was observed. The emission spectra and absolute PLQY were studied in detail in several dispersion media bringing notable arguments in favor of the PL mechanism based on the preponderant role of the surface attached functional groups and/or defects located within the carbonaceous core. Their unusual photoluminescence is especially highlighted in water where the initial blue emission is gradually shifted to intense deep green. Interestingly, when embedding the prepared CNDs in a PVA polymer matrix, the color transition was found to be reversible and dependent on water content, which is a unique feature first to be reported up to date. The recorded PLQY of 33% could recommend them for applications ranging from UV protective layers to biomedical imaging or optoelectronic devices. Also, their reversible blue to green shifting emission could be of particular interest for humidity sensors.

## Supplementary information

Supplementary information

## References

[1] Tuerhong M, Xu Y, Yin XB (2017). Review on carbon dots and their applications. Chin. J. Anal. Chem..

[2] Yao B, Huang H, Liu Y, Kang Z (2019). Carbon dots: A small conundrum. Trends Chem..

[3] Song Y (2015). Investigation from chemical structure to photoluminescent mechanism: a type of carbon dots from the pyrolysis of citric acid and an amine. J. Mater. Chem. C..

[4] Sun X, Lei Y (2017). Fluorescent carbon dots and their sensing application. TrAC Trends Anal. Chem..

[5] Boakye-Yiadom KO (2019). Carbon dots: Applications in bioimaging and theranostics. Int. J. Pharm..

[6] Kong T, Hao L, Wei Y, Cai X, Zhu B (2018). Doxorubicin conjugated carbon dots as a drug delivery system for human breast cancer therapy. Cell Prolif..

[7] Kang, Z. & Liu, Y. *Catalytic Applications of Carbon Dots. In: Carbon Nanoparticles and Nanostructures. Carbon Nanostructures*, 257–298 (Springer International Publishing, Cham, 2016).

[8] Yuan T (2019). Carbon quantum dots: an emerging material for optoelectronic applications. J. Mater. Chem. C..

[9] Zhu S (2015). The photoluminescence mechanism in carbon dots (graphene quantum dots, carbon nanodots, and polymer dots): current state and future perspective. Nano Res..

[10] Mintz KJ, Zhoua Y, Leblanc RM (2019). Recent development of carbon quantum dots regarding their optical properties, photoluminescence mechanism, and core structure. Nanoscale.

[11] Liu ML, Chen BB, Lib CM, Huang CZ (2019). carbon dots: synthesis, formation mechanism, fluorescence origin and sensing applications. Green Chem..

[12] Sciortino A, Cannizzo A, Messina F (2018). Carbon nanodots: A review—from the current understanding of the fundamental photophysics to the full control of the optical response. J. Carbon Res. C..

[13] Paloncyova M, Langer M, Otyepka M (2018). Structural dynamics of carbon dots in water and n,n-dimethylformamide probed by all-atom molecular dynamics simulations. J. Chem. Theory Comput..

[14] Stan CS, Gospei (Horlescu) P, Ursu LE, Popa M, Albu C (2017). Facile preparation of highly luminescent composites by polymer embedding of carbon dots derived from n-hydroxyphthalimide. J. Mater. Sci..

[15] Kundu A (2018). Facile approach to synthesize highly fluorescent multicolor emissive carbon dots via surface functionalization for cellular imaging. J. Colloid Interface Sci..

[16] Fan Y, Yang X, Yin C, Ma C, Zhou X (2019). Blue- and green-emitting hydrophobic carbon dots: preparation, optical transition, and carbon dot-loading. Nanotechnology.

[17] Zheng J (2018). Facile and rapid synthesis of yellow-emission carbon dots for white light-emitting diodes. J. Electron. Mater..

[18] Khare P, Bhati A, Raj S, Gunture A, Sonkar SK (2018). Brightly fluorescent zinc-doped red-emitting carbon dots for the sunlight-induced photoreduction of cr(vi) to cr(iii). ACS Omega.

[19] Liu C (2018). Orange, yellow and blue luminescent carbon dots controlled by surface state for multicolor cellular imaging, light emission and illumination. Microchim Acta..

[20] Kargbo O, Jin Y, Ding SN (2015). Recent advances in luminescent carbon dots. Curr. Anal. Chem..

[21] Choi Y, Choi Y, Kwon OH, Kim BS (2018). Carbon dots: Bottom-up syntheses, properties, and light-harvesting applications. Chem Asian J..

[22] Atabaev TS (2018). Doped carbon dots for sensing and bioimaging applications: A minireview. Nanomater. (Basel).

[23] Liu X (2017). Doped carbon dots: green and efficient synthesis on a large-scale and their application in fluorescent ph sensing. New J. Chem..

[24] Chen Z, Wang S, Yang X (2018). Phosphorus-doped carbon dots for sensing both au (iii) and l-methionine. J. Photochem. Photobiol. A: Chem..

[25] Naik VM (2018). Quick and low cost synthesis of sulphur doped carbon dots by simple acidic carbonization of sucrose for the detection of fe3+ ions in highly acidic environment. Diam. Relat. Mater..

[26] Zhao X (2019). A magnetofluorescent boron-doped carbon dots as a metal-free bimodal probe. Talanta.

[27] Xu Q (2018). Photoluminescence mechanism and applications of zn-doped carbon dots. RSC Adv..

[28] Sun S (2019). Highly luminescence manganese doped carbon dots. Chin. Chem. Lett..

[29] Stan CS, Albu C, Coroaba A, Popa M, Sutiman D (2015). One step synthesis of fluorescent carbon dots through pyrolysis of n-hydroxysuccinimide. J. Mater. Chem. C.

[30] Sanchez M, Sabio L, Galvez N, Capdevila M, Dominguez-Vera JM (2017). Iron chemistry at the service of life. IUBMB Life.

[31] Crudu M, Sibiescu D, Gurau D, Vasilescu AM (2015). New coordination compounds of fe(iii) with ligand from nhydroxysuccinimide, with applications in ecologic leather tanning technologies. Revista de Chimie Buchar..

[32] Krishnakumar V, Sivasubramanian M, Muthunatesan S (2009). Density functional theory study and vibrational analysis of ft-ir and ft-raman spectra of n-hydroxyphthalimide. J. Raman Spectrosc..

[33] Sadek, P. C. *The HPLC Solvent Guide. Second Edition* (American Chemical Society, 2002).

[34] Hill S, Galan MC (2017). Fluorescent carbon dots from mono- and polysaccharides: synthesis, properties and applications. Beilstein J. Org. Chem..

[35] Papaioannou N (2018). Structure and solvents effects on the optical properties of sugar-derived carbon nanodots. Sci. Reports.

[36] Jiang Z (2017). Understanding the photoluminescence mechanism of carbon dots. MRS Adv..

[37] van Dam B (2017). Excitation-dependent photoluminescence from single-carbon dots. Small.

[38] Singh AK, Das S, Karmakar A, Kumar A, Datta A (2018). Solvation and hydrogen bonding aided efficient nonradiative deactivation of polar excited state of 5-aminoquinoline. Phys. Chem. Chem. Phys..

[39] Dobretsov GE, Syrejschikova TI, Smolina NV (2014). On mechanisms of fluorescence quenching by water. Biophysics.

[40] Fedorenko EV (2019). The influence of the polymer matrix on luminescent properties of compositions doped with boron chelates. Opt. Spectrosc..

[41] Amato F (2019). Nitrogen-doped carbon nanodots/pmma nanocomposites for solar cells applications. Chem. Eng. Transactions.

[42] Wang X (2009). Photoinduced electron transfers with carbon dots. Chem. Commun..

[43] Li D (2019). Adverse effects of fluorescent carbon dots from canned yellow croaker on cellular respiration and glycolysis. Food Funct..

[44] Lakowicz, J. R. *Principles of Fluorescence Spectroscopy*, 97–155 (Springer, Boston, M. A, 2006).

