# Supplementary material for: Fe(III) doped carbon nanodots with intense green photoluminescence and dispersion medium dependent emission
Source: Sci Rep. 2019 Dec 11;9:18893. doi: 10.1038/s41598-019-55264-x (PMC6906313; doi:10.1038/s41598-019-55264-x)
Supplement: Supplementary file 1 — Supplementary information [file 41598_2019_55264_MOESM1_ESM.pdf]

Supplementary material

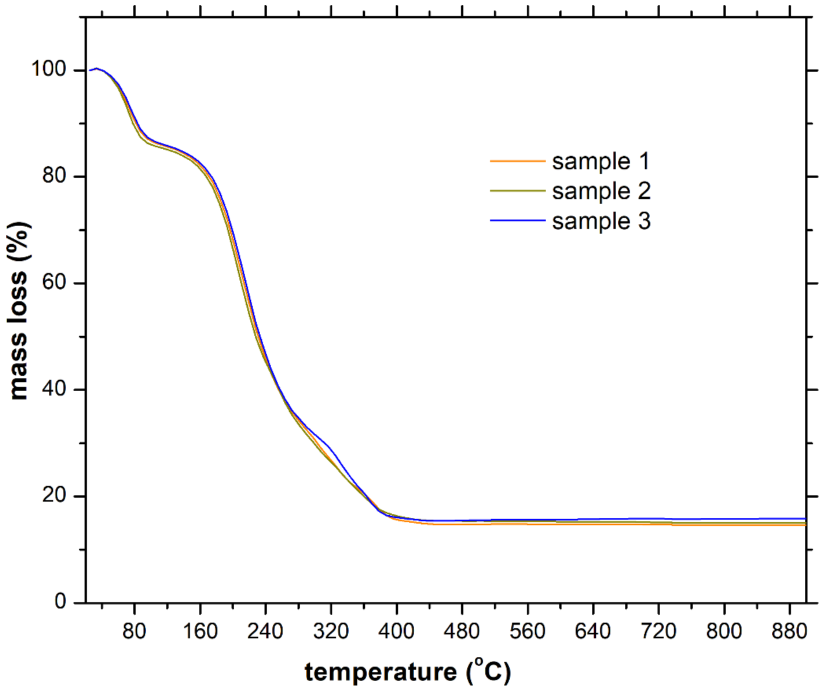

Figure S1. Mass loss recorded at TG investigation

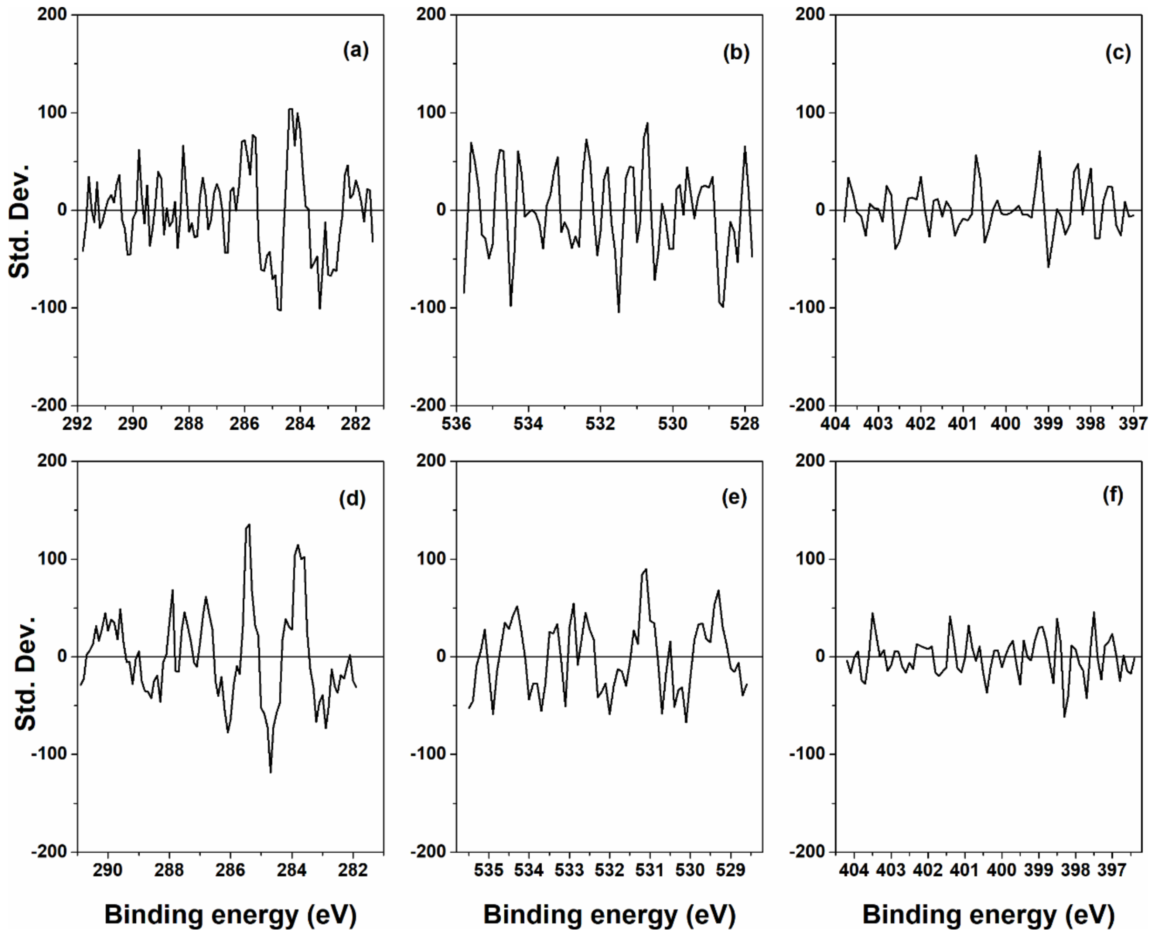

Figure S2. Fit residual plots recorded at XPS investigation

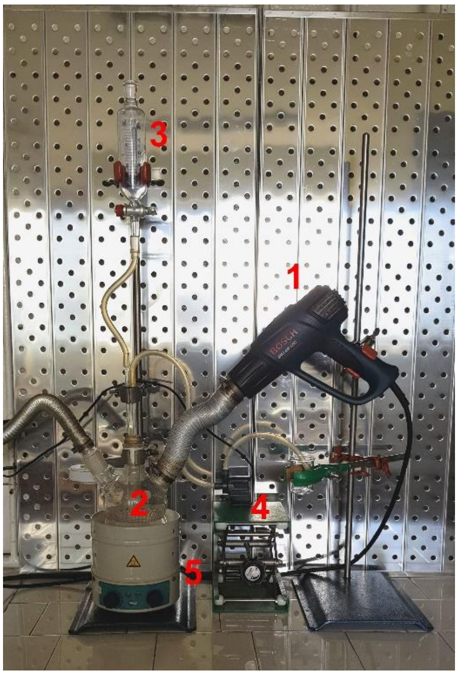

1. Temperature/flow controlled hot air source
2. Quartz tube with external glass mantle
3. Cooled water container for flooding the resulted Fe(III) doped Carbon Dots
4. Evacuation pump
5. Magnetic stirrer

Figure S3. Experimental setup for the preparation of Fe(III) doped Carbon Dots through controlled pyrolysis

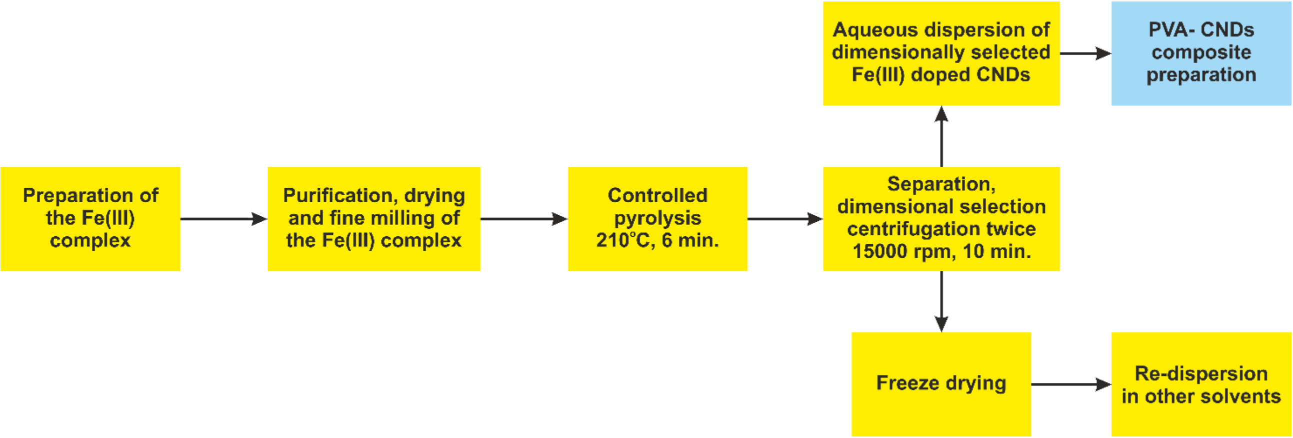

Figure S4. Preparation stages flowchart

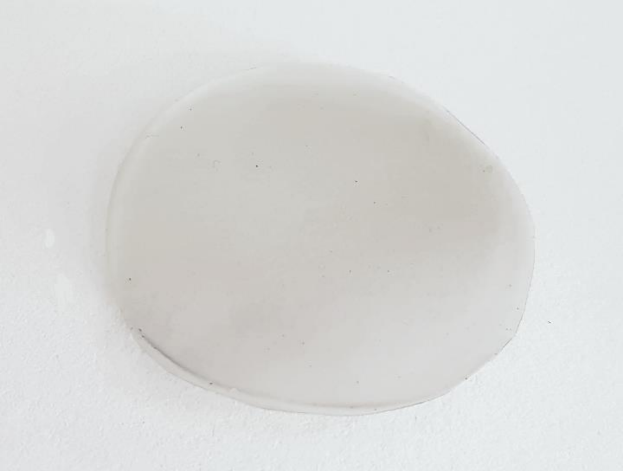

Figure S5. PVA-Fe(III) doped CNDs composite under ambient light conditions

Table S1. Recorded significant specific vibration peaks

Description	Recorded peaks (cm^{-1})		
	**N-Hydroxyphtalimide**	**[Fe(NHF)$_3$H$_2$O)]**	**Fe(III) doped CDots**
OH stretch	3476	-	-
C-H stretch	3140	3145	3209
C=C stretch	2959/2847	3036/2910	3062/2925/2854
C-C stretch	1854/1607/1383/1288	1790/1610/1376/1285	1774//1604/1386/1288
C=O sym. stretch	1784	1740	1749
C=O asym. stretch	1711	1709	1701
C-H deformation	1607	1610	1604
N-O stretch	1468/473	1465/476	1467/473
C-N stretch	1180	1188	1140
succinic ring deformation	968	975	975 (barely visible)
C=O deformation	874	881	-
succinic ring	696	700	717
aromatic ring bending	517	523	533
Fe-O- stretch	-	421	422 (barely visible)

Table S2. Three decay components and their ratios for the fluorescence lifetime of the Fe(III) doped CNDs

**τ_1 (ns)**	**A_1 (%)**	**τ_2 (ns)**	**A_2 (%)**	**τ_3 (ns)**	**A_3 (%)**	**$\langle\tau_F\rangle$ (ns)**
3.75 (St. Dev. 0.076 ns)	13.37	0.25 (St. Dev. 0.036 ns)	2.37	11.8 (St. Dev. 0.016 ns)	84.26	11.41
